# Lupin as Ingredient in Durum Wheat Breadmaking: Physicochemical Properties of Flour Blends and Bread Quality

**DOI:** 10.3390/foods13050807

**Published:** 2024-03-06

**Authors:** Alfio Spina, Carmine Summo, Nicolina Timpanaro, Michele Canale, Rosalia Sanfilippo, Margherita Amenta, Maria Concetta Strano, Maria Allegra, Martina Papa, Antonella Pasqualone

**Affiliations:** 1 Research Centre for Cereal and Industrial Crops, Council for Agricultural Research and Economics (CREA), Corso Savoia, 190, 95024 Acireale, Italy; michele.canale@crea.gov.it (M.C.); rosalia.sanfilippo@crea.gov.it (R.S.); 2 Department of Soil, Plant and Food Sciences, University of Bari Aldo Moro, Via Amendola, 165/A, 70126 Bari, Italy; carmine.summo@uniba.it; 3 Research Centre for Olive, Fruit and Citrus Crops, Council for Agricultural Research and Economics (CREA), Corso Savoia, 190, 95024 Acireale, Italy; nicolina.timpanaro@crea.gov.it (N.T.); margherita.amenta@crea.gov.it (M.A.); mariaconcetta.strano@crea.gov.it (M.C.S.); maria.allegra@crea.gov.it (M.A.); martinapapa21@gmail.com (M.P.)

**Keywords:** antioxidant activity, durum wheat bread, leavening rate, *Lupinus albus* and *L. angustifolius* flours, polyphenols, protein concentrate, rheological tests, quality, sensory profile, water and oil absorption

## Abstract

The popularity of adding pulse flours to baked goods is growing rapidly due to their recognised health benefits. In this study, increasing amounts (3, 7, 10, and 15%) of white lupin flour (*Lupinus albus* L.) and of protein concentrate from narrow-leaved lupin (*Lupinus angustifolius* L.) were used as replacements for durum wheat semolina to prepare bread, and their effects on the physicochemical properties of the flour blends, as well as the technological and sensory qualities of bread, were evaluated. The addition of protein concentrate from narrow-leaved lupin and white lupin flour increased the water binding capacity and the leavening rate compared to pure semolina. A farinograph test indicated that the dough development time had a slight but significant tendency to increase with the addition of lupin flour and protein concentrate of narrow-leaved lupin, while had a negative effect on the stability of dough. The alveograph strength decreased (225, 108, and 76 × 10^−4^ J for dough made with semolina, 15% of protein concentrate from narrow-leaved lupin, and 15% of white lupin flour, respectively), whereas there was an upward trend in the P/L ratio. Compared to re-milled semolina, the samples with lupin flour and protein concentrate from narrow-leaved lupin had low amylase activity, with falling number values ranging from 439 s to 566 s. The addition of the two different lupin flours lowered the specific volumes of the breads (2.85, 2.39, and 1.93 cm^3^/g for bread made from semolina, from 15% of protein concentrate from narrow-leaved lupin, and from 15% of white lupin flour, respectively) and increased their hardness values (up to 21.34 N in the bread with 15% of protein concentrate from narrow-leaved lupin). The porosity of the loaves was diminished with the addition of the two lupin flours (range of 5–8). The sensory analysis showed that the addition of white lupin flour or protein concentrate from narrow-leaved lupin did not impart any unpleasant flavours or odours to the bread. To conclude, the use of lupin in breadmaking requires adjustments to strengthen the gluten network but does not require a deflavouring process.

## 1. Introduction

The demand for cereal-based products that are rich in bioactive compounds (phenolics, carotenoids) and fibres is currently growing. These food products can be prepared by incorporating flours derived from pulses or vegetables [1,2,3]. The use of pulse flours, alone or mixed with wheat flours, in the production of pasta and bakery products has particularly increased in recent years [4,5,6,7]. The addition of pulse flours, in particular, represents a valuable strategy to improve the nutritional and nutraceutical values of bread [1,8,9,10,11].

Among pulses, lupin is one of the most interesting as, in addition to its high contents of dietary fibres and proteins [12,13] it has a low glycaemic impact. The latter is due to its relatively low starch content combined with the presence of γ-conglutin, an insulin-mimetic protein capable of lowering the glycaemic index [6]. Furthermore, lupin contains various bioactive peptides [14], carotenoids, tocopherols, phenolics, and phytosterols [15,16]. Lupin is also rich in dietary fibres, both soluble and insoluble, which prevent constipation and help maintain normal blood levels of cholesterol and glucose [17]. Furthermore, lupin is known to be a source of several minerals [18].

Bread, in all of its forms, is a staple food in many countries. This makes it an ideal recipient for fortification and a potential carrier of vitamins, minerals, and even phenolic compounds, presumably manifesting antioxidant activity. In this sense, the biofortification of breads should play a central role in scientific research aimed at formulating foods rich in bioactive compounds that are able to exert positive effects on human health. Bioavailability not only depends on the features of a food, but also on the factors related to an individual’s gastro-intestinal digestion. The sum of these two aspects defines the bioefficacy of food. Employing lupin as flour enriches bread with fibre and essential amino acids, including leucine, lysine, and valine, and provides considerable supplies of manganese, potassium, and phosphorus.

Villarino et al. [19] evaluated the effect of different sweet narrow-leaved lupin varieties, highlighting that the genotype has an influence on the technological characteristics of wheat bread. Pleming et al. [20] optimised the quality of wheat bread enriched with narrow-leaved lupin flour (20%) by adding vital gluten.

However, no studies have evaluated the effect of adding lupin flour to re-milled durum wheat semolina for breadmaking purposes. Re-milled semolina is widely used for breadmaking in the Mediterranean area. Durum wheat bread is particularly popular in Italy [21], where it is appreciated as a traditional and high-quality product [22,23] with a good market share.

The main aim of the present study was, therefore, to improve the contents of bioactive compounds and the antioxidant activity of durum wheat bread by partially replacing re-milled semolina with white lupin flour or with narrow-leaved lupin protein concentrate. An evaluation of the effects of supplementation on the physicochemical and rheological properties of the flour blends and doughs was conducted, in addition to technological and sensory evaluations of the breads.

## 2. Materials and Methods

### 2.1. Flours

Re-milled semolina of durum wheat [(*Triticum turgidum* L. subsp. *durum* Desf.) Husnot] (indicated as SC), with the same quality level required to prepare the “Pagnotta del Dittaino” Protected Designation of Origin (PDO) bread [23], was provided by Società Cooperativa Agricola “Valle del Dittaino” a.r.l. (Assoro, Enna, Italy). Sweet white lupin (*Lupinus albus* L.) flour (indicated as LF) was purchased from Terrena (Martigné-Ferchaud, France). Narrow-leaved lupin (*Lupinus angustifolius* L.) protein concentrate (indicated as LPC) was purchased from L.I. Frank (Twello, the Netherlands). The protein content and amino acid composition of the latter are available in [24]. The flour blends were prepared by replacing part of the re-milled semolina with increasing proportions (3, 7, 10, and 15 g/100 g) of lupin flour or lupin protein concentrate.

Particle size of re-milled semolina was in the range of 160–200 µm for 70% of particles, LPC had 95% of particles that were <100 µm in size, while LF showed 89% of particles in the range of 280–400 µm [12].

The range of replacement considered has been defined by preliminary trials, since at levels < 3%, there were no significant differences compared to SC, while above 15%, excessive increases in the ash content and brown index occurred.

### 2.2. Content of Phenolic Compounds and Antioxidant Activity

The content of phenolic compounds was assessed for both flours and breads by following the Folin–Ciocalteu colorimetric method [25]. Bread crumb was previously lyophilised (Buchi, Cornaredo, Italy) and then ground with an electric grinder (Moulinex, Ecully, France). Specifically, 5 g of flour or pulverised bread crumbs were extracted using 25 mL of a 70:30 ratio of ethanol/water solution and analysed as described in [26]. Data were expressed as gallic acid equivalents (mg GAE/100 g).

The in vitro antioxidant activity was determined according to the ORAC assay, as described by Cao et al. [27] and Ou et al. [28]. Data were reported as µmoles of Trolox equivalents (TEs).

Analyses were conducted in triplicate.

### 2.3. Water Binding Capacity and Oil Binding Capacity

The water binding capacity (WBC) and the oil binding capacity (OBC) were determined according to the method described by Sanfilippo et al. [29].

The analyses were carried out in triplicate.

### 2.4. Rheological Tests and Falling Number

A farinograph (Brabender, Duisburg, Germany) was used to determine water absorption capacity, dough development time, dough stability, and degree of softening according to the AACC method 54–21 [30].

The doughs were tested for deformation energy (W) and tenacity/extensibility ratio (P/L) according to the UNI 10453 method [31] by means of an alveograph equipped with the Alveolink ng software V1.04/99 (Tripette et Renaud, Chopin Technologies, Villeneuve-la-Garenne, France).

Falling number was measured using the falling number 1500 apparatus (Perten Instruments AB, Huddinge, Sweden) according to ISO 3093 [32].

The analyses were carried out in triplicate, except for the alveograph test, which was performed with five replications.

### 2.5. Leavening Test

The dough was prepared by kneading re-milled semolina, pure or partially replaced with different percentages (3, 7, 10, and 15 g/100 g) of LPC or LF, with 3 g/100 g of dehydrated yeast (Pizza bella alta Paneangeli, Cameo S.p.a., Desenzano del Garda, Italy), and the amount of distilled water (35 °C) was indicated by a farinograph in order to obtain a dough consistency of 500 Brabender Units (B.U.), according to the method described by Canale et al. [33].

The equation used for the leavening rate (LR) was as follows:

$$LR=\frac{\left(V-V_{0}\right)}{V_{0}}\times 100$$

where

V is the volume measured after *n* minutes;

V_0_ is the initial volume at time 0.

The analyses were carried out in triplicate.

### 2.6. Baking Test

Bread was prepared according to the AACC 10-10.03 method [30], modified as described by Ficco et al. [34]. The following physical characteristics were determined for the obtained loaves: volume, height, weight, porosity, and colour. Volume was determined using a loaf volumeter according to AACC method no. 10-05 [30]. Height was measured by using a digital slide calliper (Digi-Max^TM^, Bel-Art SP Scienceware, Wayne, NJ, USA). Weight was measured by using the Adventurer pro AV2102C digital scale (maximum capacity of 2.100 g, readability of 0.01 g; OHAUS, Pine Brook, NJ, USA). The porosity of the central slices of each bread loaf was determined by visual comparison with the eight Dallmann reference images [35] and expressed on the 8-degree Mohs–Dallmann scale [36], where 1 indicates a non-uniform structure with large and irregular cells, and 8 indicates a compact uniform structure with small and regular cells. All of the analyses were performed in triplicate.

### 2.7. Colour Determination

The colour of bread (crust and crumb separately) was assessed by using a colorimeter (CR 200, Minolta, Osaka, Japan). The CIELab colorimetric model was adopted to measure the L* (luminosity), a* (from green to red), and b* (from blue to yellow) colour coordinates. Based on the L* parameter, the brown index was calculated as follows:

Brown index = 100 − L*

This parameter provides a description of the darkness level, ranging from 0 to 100.

The total colour difference (∆E) was calculated to compare SC bread and breads with added LF and LPC according to the following equation:

$$∆E={\left[(L^{*}-L_{0}^{*}{)}^{2}+(a^{*}-a_{0}^{*}{)}^{2}+(b^{*}-b_{0}^{*}{)}^{2}\right]}^{1/2}$$

where $L_{0}^{*}$, $a_{0}^{*}$, and $b_{0}^{*}$ are the color coordinates for the reference bread (SC), whereas L*, b*, and a* are the colour coordinates of the other samples. The mean values were considered in the calculation. The obtained results were then evaluated according to the following ΔE scale: 0–2.0 = no difference; 2.0–3.5 = difference perceivable by an experienced observer; >3.5 = obvious difference.

### 2.8. Preparation of Breads for Sensory Analysis

The recipe for each type of bread is displayed in Table 1. Re-milled semolina (SC), pure or partially substituted with LF and LPC, along with yeast (Lievital, Lesaffre Italia spa, Parma, Italy), sunflower oil (Penny market srl, Scandicci, Italy), salt (Sosalt spa, Trapani, Italy), and water (by farinograph absorption at 500 B.U.) were mixed in an experimental mixer (National Manufacturing Co., Lincoln, NE, USA) at 25 °C for 5 min. The dough was left to rise in a thermostatic chamber (Giorik, Sedico, Italy) equipped with a steam humidifier (SD/SD series, Carel, Brugine, Italy) at 32–35 °C with 75–80% RH for 90 min, divided, and then placed in metal baking pans (7 × 18 × 5 cm) and allowed to rise again for 90 min under the same conditions. Baking was performed in an electric oven (Giorik, Sedico, Italy) for 7 min at 215 ± 5 °C, followed by 33 min at 165 ± 5 °C.

### 2.9. Descriptive Sensory Analysis

The ISO 13299:2016 standard [37] was followed for the sensory analysis, which was performed by a trained panel [38] of fifteen judges (7 males and 8 females, aged 28–55 y). The analysis was conducted at the CREA’s sensory laboratory (Acireale, Italy), established in accordance with the ISO 8589:2014 standard [39], and equipped with specific software for the acquisition and processing of sensory data (Smart Sensory box, Smart Sensory Solutions s.r.l., Sassari, Italy). For the application of the sensory profile method, trained judges are required to be able to characterise bread using qualitative and quantitative descriptors. Aside from terminology development, the use of reference materials is essential for efficient training, aiding in describing the various sensations and enabling an effective learning process. Assessors were trained for four months, and 22 sensory descriptors were defined with their respective references, as reported in the “Atlante Sensoriale dei Prodotti Alimentari” [40]. Descriptors were specific in terms of (i) appearance (for crumb: colour, hardness, elasticity, porosity, and regularity of porosity; for crust: crunchiness and thickness); (ii) odour (bread, yeast, roasted, and off-odour); (iii) taste (sweetness, saltiness, sourness, and bitterness); (iv) flavour (bread, yeast, roasted, and off-flavour); and (v) masticatory features (crumb moisture, chewiness, and solubility). Each judge evaluated the intensity of the sensory descriptors based on a response scale ranging from 1 (very low) to 9 (very intense). A slice of each bread sample, comprising crust and crumb, cut approximately 10 min before tasting and about 1.5 cm thick, was presented to the judges at room temperature on a plastic plate labelled with three-digit codes. The codes were automatically produced by the smart sensory box. The sample presentation was balanced and randomised. Still mineral water was provided to the panellists to clean their palates between evaluations.

### 2.10. Statistical Analyses

All data (mean ± standard deviation) were subjected to a one-way analysis of variance (ANOVA) by using the Statgraphics^®^ Centurion XVI (Statpoint Technologies, The Plains, VA, USA) software. The difference between the means was determined using the Tukey test at the probability level of *p* ≤ 0.001 for all of the parameters, with the exception of the red index of crust (*p* ≤ 0.01), falling number, and brown index of crumb (*p* ≤ 0.05). The correlation coefficient (r) between the content of phenolic compounds and antioxidant activity was calculated.

Two multivariate techniques were used sequentially, namely the Hierarchical Cluster Analysis and the K-means cluster analysis, on the dataset of all of the variables studied in flours and breads. The Hierarchical Cluster Analysis was preceded by pretreatment of the variables through the calculation of standardised scores (z-score variables). This initial step aimed to identify meaningful patterns and determine the optimal number of clusters within the extensive dataset without a priori information.

Subsequently, the K-means cluster analysis was performed by utilising the number of clusters identified in the Hierarchical Cluster Analysis as input for its algorithm. This step aimed to assign and interpret cluster memberships effectively through the qualitative–quantitative footprint generated using the analysis. This statistical analysis was conducted with the IBM SPSS Statistics software version 20 (IBM Corporation, 2011, Armonk, NY, USA).

## 3. Results and Discussion

### 3.1. Phenolic Compounds and Antioxidant Activity in Pure Flours and Their Blends

The total phenolic compounds and antioxidant activity, the latter determined according to the ORAC assay, are shown in Table 2.

The results for the total Folin–Ciocalteu reducing capacity, reported as phenolic compounds, showed significantly (*p* ≤ 0.001) higher values in the LPC (37.91 mg GAE/100 g) and LF (33.58 mg GAE/100 g) than in SC (21.88 mg GAE/100 g). Particularly relevant was the antioxidant activity of 76.33 µmol TE/g d.m. observed in the LPC, as well as the value of 61.00 µmol TE/g d.m. assessed in LF, which are both significantly higher (*p* ≤ 0.001) than the value determined in SC. However, no statistical difference was observed among the flour blends. As for the antioxidant activity, Ferchichi et al. [41] found higher values but performed the ABTS test. A correlation (*p* ≤ 0.001; r = 0.59) was observed between the contents of phenolic compounds and antioxidant activity, as also reported in other studies [42].

### 3.2. Water Binding and Oil Binding Capacities of Flour Blends

**Figure 1:**
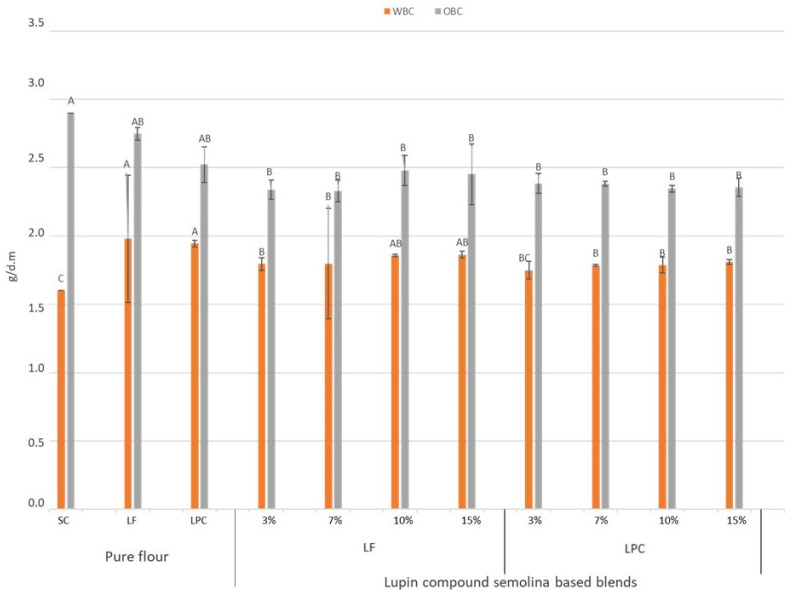
Water binding capacity (WBC) and oil binding capacity (OBC) of re-milled semolina (SC), lupin flour (LF), protein concentrate from narrow-leaved lupin (LPC), and their blends, prepared with increasing levels of replacement (3, 7, 10, and 15 g/100 g). Different letters indicate significant difference (*p* ≤ 0.001).

The water binding capacity of the control was lower than that for LF. The latter, indeed, showed a particularly high value for this parameter (4.41 g H_2_O/g d.m.). The LPC, instead, accounted for 1.94 g H_2_O/g d.m. and did not show a statistically significant difference with SC and with the flour blends (Figure 1). Pasarin et al. [43] observed lower WBC values for the lupin protein isolate, while similar values for both the protein isolate and lupin flours were reported by Khalid and Elharadallou [44].

The particularly high value of water binding capacity observed in LF was imputable to its contents of proteins and fibres and its ability to establish hydrogen bonds with water, and was also due to its particle size (<100 µm), which was smaller than that of SC. From a practical point of view, a high water binding capacity value is interesting because it could lead to a higher dough weight [45,46,47]. High water binding capacity values in pulses were assessed by other authors [48,49,50]. Specifically, some authors argued that the high water binding capacity of pulse proteins is due to the presence of a great number of hydroxyl groups [51,52]. Pulse proteins can compete for water with other constituents of the dough system, which results in high water absorption, as assessed using a farinograph [53,54,55].

The oil binding capacity was significantly higher in SC (2.90 g oil/g d.m.) than in all of the flour blends containing LPC, as well as those with LF at 3% and 7%. Other authors [45] found lower oil binding capacity values in lupin flour (1.76 g oil/g), but found similar values in lupin protein isolate (2.80 g oil/g).

The oil binding capacity is an important property for the bakery industry, as it is related to the emulsifying capacity. Several factors influence this parameter, including, for example, the composition of the protein fraction. Proteins mostly influence the oil binding capacity since non-polar amino acid side chains can establish hydrophobic interactions with the hydrocarbon chains of lipids [56,57,58,59,60]. Flours with high oil binding capacity values retain flavour and improve mouthfeel when used in foods [61]. The observed values of water and oil binding capacities were like those reported for flour from other pulse species [62], confirming a higher oil-absorbing capacity compared to water for both samples.

### 3.3. Rheological Testing of Dough and Falling Number

The results of the farinograph and alveograph tests are reported in Table 3.

Water absorption, determined using a farinograph, increased as the level of replacement of SC increased. In detail, the higher the percentage of LF, the greater the water absorption, which is in agreement with the findings of other authors [54,63,64,65], due to the water binding properties of fibre. The addition of LPC induced an increase in the absorption of water at a lower extent compared to LF, which was probably due to lower content of fibre [12]. Lupin protein concentrate, indeed derived from a process of enrichment in proteins, with reduction of other macronutrients..

The farinograph test showed that SC had a short dough development time, coupled with a prolonged stability of the dough to kneading and, thus, a low softening index. The dough development time showed a slight but significant tendency to become longer with the addition of LF and LPC due to the higher fibre content, which is known to interfere with dough formation and slow it down. Indeed, the fastest dough development time was observed in SC (1.36 min), while the longest was observed in LPC 15% (3.08 min), with significant differences among the flour blends.

In agreement with the dough development time data, the stability of the dough was negatively affected by the addition of LF and LPC, at an extent that was higher for LF than for LPC, as supposed. The former, at the highest level of supplementation (15 g/100 g), caused the most detrimental effect: it shortened the stability to 1.83 min, which is almost three-times lower (5.23 min in SC). The farinograph stability indicates the dough’s ability to withstand intense mechanical stress, such as prolonged kneading, relying on the strength of the gluten network. The addition of lupin, a gluten-free species, was expected to worsen the dough’s properties, particularly in LF, which had more fibres and fewer proteins than LPC. Dervas et al. [53] observed a more prolonged stability when adding lupin flour at 5 g/100 g, but Doxastakis et al. [54], Villacrés et al. [62], and López [66] found a shorter stability as the level of addition of lupin flour increased.

The degree of dough softening, which is known to be inversely correlated with farinograph stability, was low in the case of SC (45 B.U.) and significantly increased when adding LF and LPC, with a particularly negative effect at the highest level of addition (15 g/100 g) for both (119 B.U. with 15% of LPC and 107 B.U. with 15% of LF). The negative effect of fibre and the absence of gluten in lupin flour products may explain these results.

As for the alveograph test, the highest value of deformation energy (alveograph W) was observed in SC (225 × 10^−4^ J), while the lowest was observed in LF 15% (76 × 10^−4^ J). The deformation energy for the flour with 15% of LPC was characterised by a significantly higher value than that for 15% of LF, accounting for 108 × 10^−4^ J. The deformation energy was affected by the addition of LPC and, to a greater extent, by the addition of LF, due to the presence of more fibre in the latter [12].

The tenacity-to-extensibility (P/L) ratio was very high for all of the samples. The SC showed a value of 2.40, which tended to increase with the addition of LF (3.94 for 15% of LF) and LPC (3.49 for 15% of LPC). However, only the difference between SC and 15% of LF was statistically significant (*p* < 0.001). The high tenacity-to-extensibility ratio observed in SC is an intrinsic characteristic of durum wheat milling products [23].

Strong gluten and balanced visco-elastic properties are both required for optimal loaf development. Therefore, the worsening of the alveograph and farinograph indices observed in the semolina blends containing LF and LPC, especially at the highest level of replacement, induce a lower breadmaking performance compared to the control.

The falling number test provides a measure of the amylase activity of flour. High values mean low amylase activity and vice versa. The partial replacement of re-milled semolina significantly affected the amylase activity of the samples, as revealed by statistically significant differences. The data in Table 3 point out a lower falling number for the re-milled semolina (419.00 s), indicating higher amylase activity compared to the lupin matrix-supplemented samples. The two lupin-enriched groups behaved similarly to each other. In both groups, there was a general decline in the falling number values as a reaction to the growing integration rate. Specifically, the highest falling number values were assessed in the 3% LF (503.50 s) and the 7% LPC samples (566.00 s), indicating lower amylase activity compared to 15% LF and 15% LPC, respectively.

### 3.4. Dough Fermentation Test

**Figure 2:**
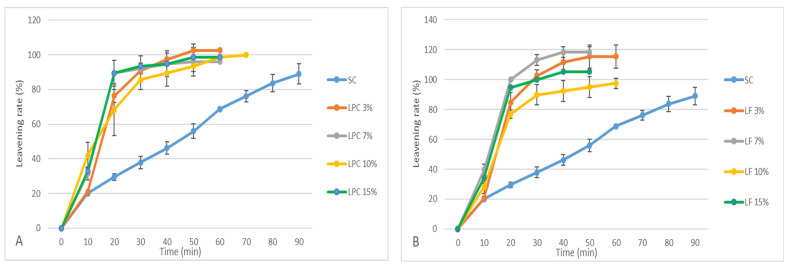
Leavening rates of doughs prepared with increasing levels of replacement (3, 7, 10, and 15 g/100 g) of re-milled semolina (SC) with narrow-leaved lupin protein concentrate (LPC; plot (**A**)) and white lupin flour (LF; plot (**B**)).

The results obtained from the leavening test reveal that the addition of LPC and LF shortened the leavening times and led to a greater expansion of the dough, resulting in an increased leavening rate compared to that of SC. In detail, the doughs with the addition of LPC (Figure 2A) reached a 103% leavening rate, while the addition of LF (Figure 2B) led to a leavening rate of 118%.

Lupin flour, in particular, was characterised by a higher leavening rate than that of LPC, which was probably due to the greater presence of fermentable fibre [12]. However, after an initial rapid expansion, these doughs could not stand long leavening times, as shown by the farinograph and alveograph data.

### 3.5. Physicochemical Characteristics of Bread

Table 4 shows the physicochemical characteristics of the bread variants. The addition of LPC (which had a limited water binding capacity) at levels higher than 10% reduced the bread’s moisture compared to SC. Similar results were observed when the addition of LF was relatively low, i.e., 3% and 7% LF.

The SC bread had the maximum height (76 mm). The addition of LF and LPC led to a reduction in height but without significant differences, except for 15% LF (60.1 mm). It is supposed that the addition of LF and LPC caused a reduction in the specific volume and, consequently, led to a denser bread crumb. The specific volume ranged from 1.93 cm^3^/g (LF 15%) to 2.85 cm^3^/g (SC) and, inversely, the density ranged from 0.35 g/cm^3^ (SC) to 0.52 g/cm^3^ (LF 15%). The differences between the SC breads were always statistically significant.

As for the bread hardness, SC showed the value of 16.85 N, which increased to 21.17 for 15% LF and 21.34 N for 15% LPC, but the differences were not significant. The porosity of the bread crumb was scored from 5 (SC) to 8 (LF 15%), with a score of 7 being attributed to 10% LPC and 15% LPC. Higher values of porosity indicated smaller cells than those with lower values.

In general, the addition of LF and LPC worsened the technological qualities of the breads, reducing the specific volumes and the heights of the loaves, increasing their hardness values, and determining a denser porosity (the latter indicated by higher scores on the Dallmann scale). These effects, already predicted by the alveograph and farinograph data, were due to gluten dilution and fibre addition, which both reduced the loaves’ ability to retain fermentation gas [5,10,20,67,68]. Therefore, the addition of lupin flour/protein concentrate to durum wheat semolina requires an adjustment that does not exclude the addition of improvers to compensate for the weakening of the gluten network.

Dervas et al. [53], instead, tested the additions of whole lupin flour, lupin concentrate, and defatted lupin concentrate to a medium-strength soft wheat flour, obtaining acceptable breads in terms of consistency and structure without significant differences in the specific volume with respect to the control when the levels of integration were ≤10%. The flour of soft wheat, indeed, having a more pronounced extensibility than durum wheat re-milled semolina, can better tolerate the addition of any gluten-diluting and fibre-rich flour. The same study, however, highlighted that the specific volume significantly decreased when greater amounts (>10%) of lupin flour or concentrate were added [53].

The addition of LPC and LF, at any percentage, determined a significant increase in density and, therefore, an improvement in the bread yield (i.e., the quantity of bread that can be produced from a given weight of flour) compared to the SC bread. This increase was imputable to the hygroscopic effect of fibre [69,70] and was more pronounced for the breads with LF (richer in fibre) than the ones with LPC. As reported in Spina et al. [12], lupin flour and lupin protein concentrate showed higher values of fibre than SC.

The contents of phenolic compounds, reported in Table 5, show a statistical difference (*p* ≤ 0.001; r = 0.74) between the breads with different amounts of LPC and LF, with values reaching 25.41 mg GAE/100 g d.m. in breads with 15% of LPC.

Compared to SC bread, a considerable rise in phenolic compounds was observed with the addition of LPC and LF, even at the lowest percentage of fortification. This positive effect was more marked than in the starting flour blends. Due to lupin flour and lupin protein concentrate being richer in fibre than re-milled semolina [12], and due to fibre being able to bind phenolic compounds, a fraction of these phenolics was probably released with fermentation. In fact, despite the fact that the intense thermal treatment of baking is known to degrade phenolic compounds, their reduction compared to the starting flour accounted for 30% in the fortified breads, while it reached 45% in the SC bread.

**Figure 3:**
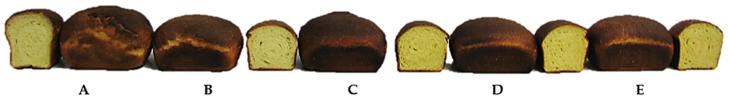
Breads prepared with replacement of re-milled semolina with flour prepared from lupin protein concentrate at increasing levels (3, 7, 10, and 15 g/100 g). From left to right: re-milled semolina 100% (**A**), LPC 3% (**B**), LPC 7% (**C**), LPC 10% (**D**), LPC 15% (**E**).

**Figure 4:**
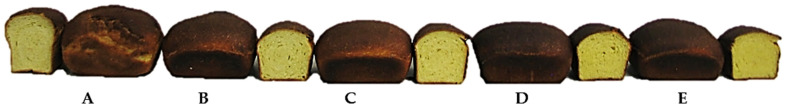
Breads prepared with replacement of re-milled semolina with lupin flour at increasing levels (3, 7, 10, and 15%). From left to right: re-milled semolina 100% (**A**), LF 3% (**B**), LF 7% (**C**), LF 10% (**D**), LF 15% (**E**).

A similar behaviour was also observed for antioxidant activity, which reached 36.00 µmol/g d.m. in the bread with 15% of LPC. Also, the antioxidant activities of the breads was lower than that of the corresponding flour blends due to the thermal degradation of the antioxidant compounds. However, the results for both the phenolics and antioxidant activity of the bread were higher than those observed by Villarino et al. [71] in bread samples obtained by adding 20% of different varieties of *L. angustifolius* to soft wheat flour. Semolina typically has a higher content of bioactive compounds than common wheat flour. This is also the reason why the values quoted were higher than those found by these authors. The evaluation of the bread’s colour was carried out separately in the crumb and crust (Table 6). As regards the crumb, the differences in the brown index among the samples were not statistically significant. The value of a* of the breads with LF was not significantly different compared to that of the SC bread, while it was significantly increased in the breads containing LPC at levels greater than 7 g/100 g. The lowest value of b* was observed in the SC bread and, again, significantly increased in the bread samples prepared by incorporating levels of LPC or LF greater than 7 g/100 g. Therefore, the crumb of the breads fortified with LPC (Figure 3) or LF (Figure 4) showed a slightly more intense (*p* < 0.001) yellow hue than the SC bread due to the relevant presence of carotenoids in lupin [72]. Carotenoids, indeed, are also contained in re-milled semolina, but at a lower extent. The observed increase in b* and the increase in a* were also reported by authors who added lupin milling products to soft wheat flour [73]. The observed differences in the individual colour coordinates are summarised in ∆E, indicating that fortification levels below 7 g/100 g did not result in clearly visible differences in the crumb colour compared with the reference bread (SC).

As regards the crust, its colour was obviously much darker than the crumb due to the Maillard reaction and caramelisation of sugars, which smoothed the difference among the samples. In fact, for both the brown index and a*, significant differences were observed between the supplemented breads and the SC bread only when high levels of lupin flour were incorporated (10% and 15% of LF). In particular, with the addition of LF, the brown index increased, while a* decreased. Also, b* decreased when comparing the SC bread to those prepared using the flour blends, with significant differences except for the bread with 3% of LPC. The ∆E values for the crust colour were lower than those for the crumb colour and still indicated that fortification levels below 7 g/100 g did not result in visible differences in the crust colour compared to that of the SC bread.

### 3.6. Sensory Characteristics of the Breads

The appearance, taste, odour, and texture of a food product are very important predictors for whether or not the product will be acceptable to consumers.

**Figure 5:**
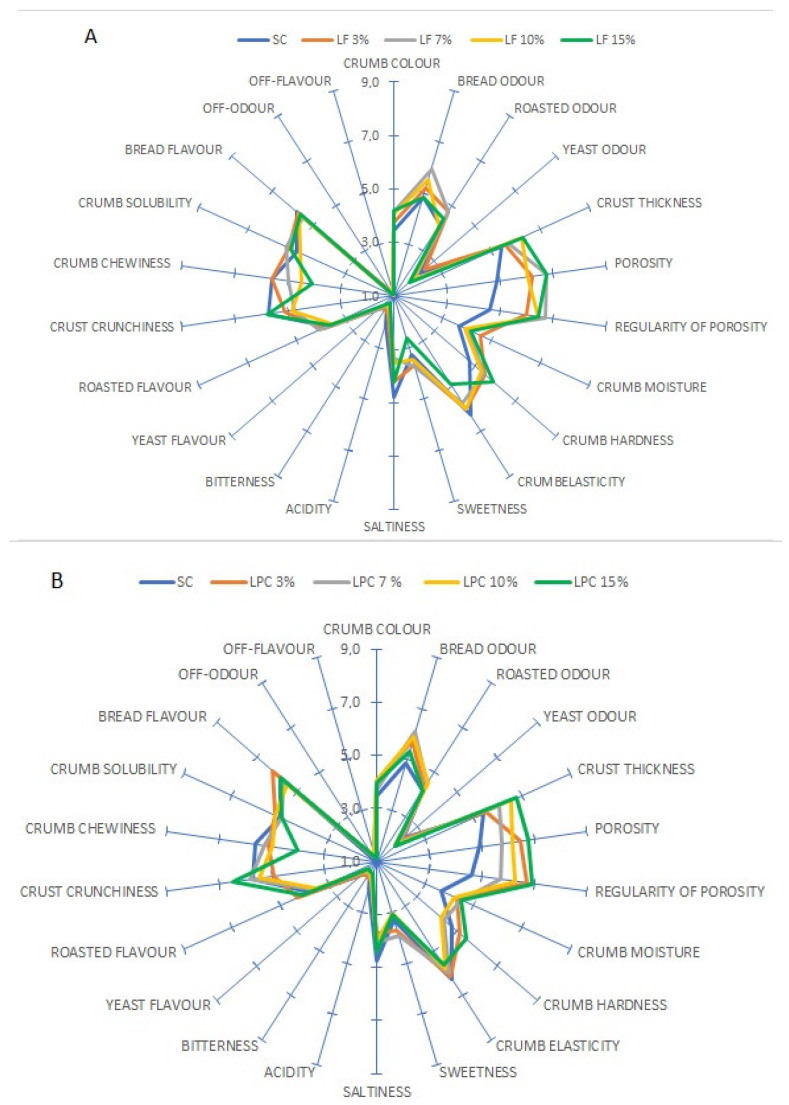
(**A**) Sensory profiles of breads prepared by replacing re-milled semolina (SC) with lupin flour (LF) at increasing levels (3, 7, 10, and 15 g/100 g). (**B**) Sensory profiles of breads prepared by replacing re-milled semolina (SC) with flour prepared from lupin protein concentrate (LPC) at increasing levels (3, 7, 10, and 15 g/100 g).

Bread samples containing LF at different concentrations differed from SC bread mainly in terms of descriptors related to visual and tactile characteristics, such as crust thickness, porosity, regularity of porosity, crumb chewiness, and crumb hardness (Figure 5A). The bread with 15% of LF markedly differed from the other samples due to its lower chewiness and greater crunchiness. The addition of LF led to a denser and less regular porosity compared to the SC bread and to a more intense crumb colour. A similar trend for these descriptors was found in the bread samples containing LPC, especially at the highest percentage (Figure 5B). Overall, the sensory evaluation was in agreement with the instrumental analysis of the bread’s physical attributes in terms of porosity, hardness, and specific volume.

All of the bread samples evaluated had no off-flavours or off-odours that could adversely affect consumer acceptance. This is an interesting observation, since the off-flavour of pulse seeds, including lupin, is a recognised issue [74]. The dilution into semolina and the emergence of a new flavour due to the chemical transformations related to baking, such as the Maillard reaction and caramelisation (especially in the crust), probably reduced any possible off-flavours below the threshold of perception.

### 3.7. Cluster Analysis (Hierarchical Cluster Analysis and K-Means Cluster Analysis) of Flours, Doughs, and Breads

**Figure 6:**
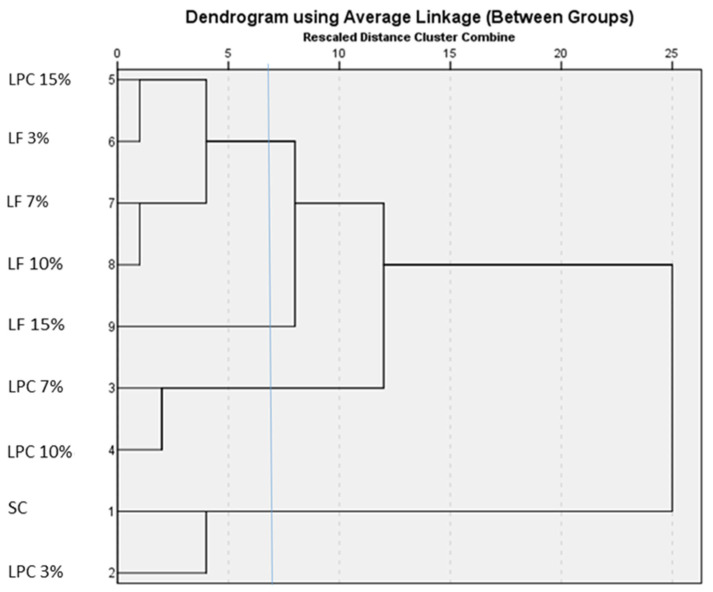
Dendrogram of Hierarchical Cluster Analysis of flours, doughs, and breads.

A Hierarchical Cluster Analysis was performed on the complete dataset of measured variables. Before the clustering procedure, standardised scores were calculated for all variables and saved as new z-score variables. This pretreatment ensured that each variable contributed equally to the distance measurements. The squared Euclidean distance measurement and the single-linkage (nearest neighbour) method of clustering were utilised (Figure 6).

A four-cluster solution identified three clusters, which were further segmented, and a single branch: 15% of LF. A first cluster included 15% of LPC and 3%, 7%, and 10% of LF. The pair of 7% and 10% of LPC was joined in a cluster, as well as the pair of SC and 3% of LPC. The four-cluster solution was the conclusion of the Hierarchical Cluster Analysis and was used as the input for the K-means cluster analysis algorithm.

The cluster membership obtained from the K-means cluster analysis is given in Supplementary Table S1. The control (SC) formed a cluster of its own (cluster 1), like 3% of LPC (cluster 2). The third cluster included the triplet 7%, 10%, and 15% of LPC; 3%, 7%, 10%, and 15% of LF were grouped in cluster 4. The distance to the cluster centre is given; the smaller the value, the closer it is to the middle of that cluster, i.e., the centroid, and the more representative that flour and/or bread is of that cluster. From our results, 10% of LPC (cluster 3) and 10% of LF (cluster 4) were the centroids of their own group.

**Figure 7:**
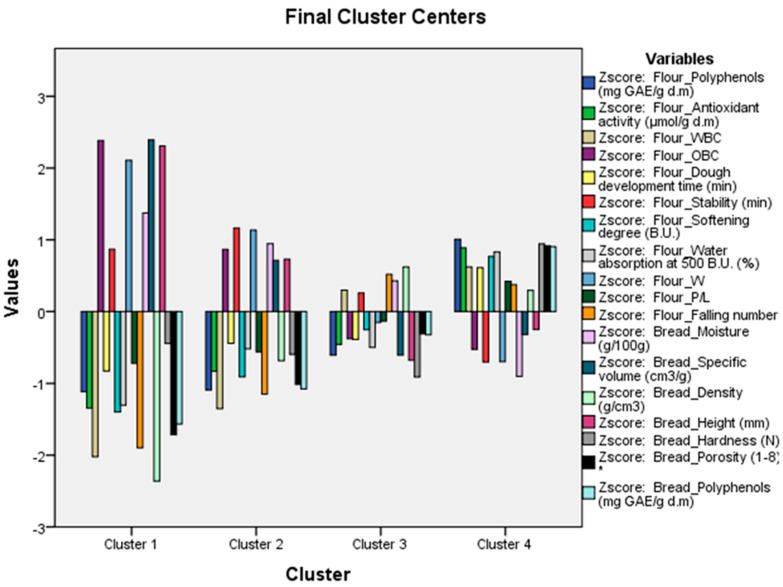
K-means cluster analysis in flours and breads: qualitative and quantitative fingerprints of each cluster based on score of each variable.

The graphical representation of the final cluster centres, based on the scores of each variable (Supplementary Table S2), which are specific to each cluster, gives us the quali–quantitative footprint of each of them (Figure 7). Cluster 1, represented exclusively by SC, showed the largest values of OBC, W of the flour, and specific volume and height of the bread. Conversely, the same cluster was characterised by particularly low values of antioxidant activity, WBC, softening degree, water absorption, falling number, polyphenols, antioxidant activity, bread density, and porosity.

## 4. Conclusions

This study showed that the addition of lupin flour or lupin protein concentrate increased the water binding capacity of flour and the leavening rate of dough compared to pure semolina. The dough’s rheological properties worsened due to gluten dilution and fibre increase. As a result, the addition of these flours caused a reduction in the specific volume of durum wheat bread and a harder crumb texture. On the other hand, the amount of polyphenols and the antioxidant activity of the flour significantly improved with the proposed fortification.

Finally, the sensory analysis showed that the addition of lupin flour or lupin protein concentrate did not impart any unpleasant flavours or odours to the bread. Therefore, the use of lupin in breadmaking, with a minimum concentration of up to 15%, does not require a deflavouring process.

## Figures and Tables

**Table 1:** Formulations of experimental breads (g for 100 g of wheat flour). SC: re-milled semolina; LF: lupin flour; LPC: protein concentrate from narrow-leaved lupin.

Bread Type	Semolina	LPC	LF	Yeast	NaCl	Sunflower Oil	Water
SC	100	-	-	0.5	1.7	3.33	62.3
LF 3%	97	-	3	0.5	1.7	3.33	65.5
LF 7%	93	-	7	0.5	1.7	3.33	66.3
LF 10%	90	-	10	0.5	1.7	3.33	66.7
LF 15%	85	-	15	0.5	1.7	3.33	69.1
LPC 3%	97	3	-	0.5	1.7	3.33	62.1
LPC 7%	93	7	-	0.5	1.7	3.33	63.8
LPC 10%	90	10	-	0.5	1.7	3.33	63.9
LPC 15%	85	15	-	0.5	1.7	3.33	64.1

**Table 2:** Contents of phenolic compounds and antioxidant activity of re-milled semolina (SC), lupin flour (LF), protein concentrate from narrow-leaved lupin (LPC), and their blends, prepared at increasing levels of replacement (3, 7, 10, and 15 g/100 g).

Sample	Polyphenols (mg GAE/100 g d.m.)	Antioxidant Activity (µmol TE/g d.m.)
*Pure flours*		
SC	21.88 ± 2.19 c	56.67 ± 0.20 b
LF	33.58 ± 0.18 ab	61.00 ± 1.06 b
LPC	37.91 ± 1.48 a	76.33 ± 1.69 a
*Blends*		
LF 3%	22.17 ± 0.16 c	56.80 ± 0.16 b
LF 7%	26.32 ± 0.11 bc	56.97 ± 0.11 b
LF 10%	27.15 ± 1.07 bc	57.11 ± 0.07 b
LF 15%	29.59 ± 0.18 ab	57.32 ± 0.01 b
LPC 3%	22.36 ± 2.08 c	57.26 ± 0.24 b
LPC 7%	23.00 ± 1.93 c	58.77 ± 0.61 b
LPC 10%	24.85 ± 0.13 c	59.35 ± 0.83 b
LPC 15%	25.91 ± 0.02 bc	60.69 ± 2.49 b

GAE: gallic acid equivalents; TE: Trolox equivalents. Data are expressed as mean ± standard deviation. Different letters in the same column indicate significant differences (*p* ≤ 0.001) based on Tukey’s HSD.

**Table 3:** Farinograph and alveograph data of re-milled semolina (SC) and of its blends with protein concentrate from narrow-leaved lupin (LPC) or with white lupin flour (LF) at different percentages (3, 7, 10, and 15 g/100 g).

Sample	Farinograph			Alveograph			Falling Number (s)
	Dough Development Time (min)	Stability (min)	Softening Degree (B.U.)	Water Absorption at 500 B.U. (%)	W (10^−4^ J)	P/L	
SC	1.36 ± 0.02 e	5.23 ± 0.05 b	45 ± 0.7 f	62.3 ± 0.4 e	225 ± 4 a	2.40 ± 0.03 bc	419 ± 14 d
LF 3%	1.62 ± 0.04 c	5.13 ± 0.40 b	62 ± 2.1 d	65.5 ± 0.1 c	114 ± 1 cd	1.77 ± 0.06 c	503 ± 20 ac
LF 7%	1.72 ± 0.03 b	4.93 ± 0.03 b	54 ± 2.8 e	66.3 ± 0.1 b	96 ± 3 ef	1.81 ± 0.13 c	487 ± 9 bcd
LF 10%	1.59 ± 0.01 c	4.87 ± 0.08 b	60 ± 2.1 e	66.7 ± 0.1 b	82 ± 1 fg	2.06 ± 0.03 bc	461 ± 8 bcd
LF 15%	1.48 ± 0.03 d	1.83 ± 0.04 e	107 ± 3.5 ab	69.1 ± 1.06 a	76 ±1 g	3.94 ± 0.06 a	439 ± 28 cd
LPC 3%	1.69 ± 0.02 b	6.05 ± 0.14 a	6 ± 7.8 d	62.1 ± 0.1 e	147 ± 1 b	2.89 ± 0.56 abc	488 ± 12 bcd
LPC 7%	1.52 ± 0.04 d	2.61 ± 0.02 d	83 ± 2.1 c	63.8 ± 1.2 d	127 ± 2 c	2.27 ± 0.15 bc	566 ± 28 a
LPC 10%	1.61 ± 0.07 c	3.83 ± 0.08 c	78 ± 7.1 cd	63.9 ± 0.1 d	122 ± 1 cd	2.49 ± 0.78 abc	530 ± 9 ab
LPC 15%	3.08 ± 0.05 a	1.72 ± 0.01 e	119 ± 6.4 a	64.1 ± 0.1 d	108 ± 3 de	3.49 ± 0.08 ab	518 ± 26 ab

W: Alveograph deformation energy; P/L: tenacity-to-extensibility ratio; B.U.: Brabender units. Different letters in columns indicate significant difference (*p* ≤ 0.001 for farinograph and alveograph; *p* ≤ 0.05 for falling number).

**Table 4:** Physicochemical characteristics of bread prepared with re-milled semolina (SC) or with blends of re-milled semolina and protein concentrate from narrow-leaved lupin (LPC) or white lupin flour (LF) at different percentages (3, 7, 10, and 15 g/100 g).

Bread Type	Moisture (g/100 g)	Specific Volume (cm^3^/g)	Density (g/cm^3^)	Height (mm)	Hardness (N)	Porosity (1–8) *
SC	29.98 ± 0.18 a	2.85 ± 0.05 a	0.35 ± 0.07 c	76.0 ± 0.0 a	16.85 ± 0.27 abc	5
LF 3%	28.64 ± 0.01 b	2.14 ± 0.05 bc	0.47 ± 0.19 ab	63.4 ± 0.7 ab	16.36 ± 0.82 c	6
LF 7%	28.83 ± 0.02 b	2.21 ± 0.03 bc	0.45 ± 0.21 b	64.1 ± 0.2 ab	16.81 ± 0.99 abc	6
LF 10%	29.22 ± 0.01 ab	2.32 ± 0.03 b	0.43 ± 0.12 b	63.1 ± 0.0 ab	18.49 ± 1.75 abc	6
LF 15%	29.22 ±0.03 ab	1.93 ± 0.02 c	0.52 ± 0.10 a	60.1 ± 1.8 b	21.17 ± 1.59 ab	8
LPC 3%	29.33 ± 0.02 ab	2.16 ± 0.01 bc	0.46 ± 0.07 ab	63.2 ± 0.1 ab	15.79 ± 0.89 c	6
LPC 7%	29.53 ± 0.01 ab	2.24 ± 0.07 bc	0.45 ± 0.07 b	65.7 ± 1.4 ab	16.65 ± 1.68 bc	6
LPC 10%	28.60 ± 0.42 b	2.19 ± 0.03 bc	0.46 ± 0.05 b	64.6 ± 0.4 ab	18.51 ± 1.36 abc	7
LPC 15%	28.41 ± 0.01 b	2.32 ± 0.01 b	0.43 ± 0.07 b	65.6 ± 0.1 ab	21.34 ± 1.64 a	7

* Value of 1: very porous; 8: little porous. Different letters in column indicate significant difference (*p* ≤ 0.001).

**Table 5:** Contents of phenolic compounds and antioxidant activity of bread prepared with re-milled semolina (SC) or with blends of re-milled semolina and protein concentrate from narrow-leaved lupin (LPC) or white lupin flour (LF) at different percentages (3, 7, 10, and 15 g/100 g).

Sample	Polyphenols (mg GAE/g d.m.)	Antioxidant Activity (µmol/g d.m.)
SC	12.08 ± 0.05 g	17.66 ± 1.34 d
LF 3%	15.36 ± 0.09 f	18.35 ± 0.47 d
LF 7%	16.30 ± 0.08 ef	21.96 ± 0.89 cd
LF 10%	17.33 ± 0.20 de	24.38 ± 0.68 c
LF 15%	18.20 ± 0.04 cd	24.47 ± 0.43 c
LPC 3%	16.26 ± 0.26 ef	26.59 ± 0.38 bc
LPC 7%	19.12 ± 0.15 c	30.67 ± 0.07 ab
LPC 10%	22.38 ± 0.02 b	31.05 ± 0.29 ab
LPC 15%	25.41 ± 0.30 a	36.00 ± 1.05 a

Data are expressed as mean ± standard deviation. Values in column indicated by different letters are significantly different (*p* ≤ 0.001) based on Tukey’s HSD.

**Table 6:** Colour characteristics of breads prepared with re-milled semolina (SC) or with blends of re-milled semolina and protein concentrate from narrow-leaved lupin (LPC) or white lupin flour (LF) at different percentages (3, 7, 10, and 15 g/100 g).

Bread Type	Crumb				Crust			
	Brown Index (100 − L*)	Red Index (a*)	Yellow Index (b*)	∆E	Brown Index (100 − L*)	Red Index (a*)	Yellow Index (b*)	∆E
SC	25.8 ± 0.1 ab	−3.0 ± 0.1 d	21.8 ± 0.3 e	-	64.0 ± 1.2 c	12.4 ± 0.1 a	17.0 ± 0.6 a	-
LF 3%	27.0 ± 0.1 ab	−3.0 ± 0.1 d	26.1 ± 1.6 cde	3.9	64.8 ± 1.3 bc	9.7 ± 0.4 abc	14.1 ± 0.1 b	3.7
LF 7%	25.8 ± 0.0 ab	−3.1 ± 0.1 d	28.3 ± 0.8 bcd	6.1	65.7 ± 0.3 bc	9.7 ± 0.0 abc	13.0 ± 0.9 bc	4.9
LF 10%	29.6 ± 0.0 ab	−3.0 ± 0.1 d	32.6 ± 1.0 ab	11.7	70.4 ± 1.4 a	7.0 ± 0.0 c	8.1 ± 0.1 d	10.9
LF 15%	27.3 ± 0.0 ab	−2.8 ± 0.2 cd	36.4 ± 1.8 a	14.1	68.5 ± 0.7 ab	8.5 ± 0.0 bc	11.1 ± 0.4 c	8.7
LPC 3%	25.3 ± 0.0 b	−2.1 ± 0.1 bcd	23.0 ± 1.6 de	1.7	62.8 ± 2.2 c	10.4 ± 0.3 ab	15.3 ± 1.7 ab	2.1
LPC 7%	28.9 ± 0.1 ab	−1.5 ± 0.2 bc	31.0 ± 1.7 abc	7.7	67.0 ± 0.9 abc	8.7 ± 0.0 bc	11.2 ± 0.9 c	4.2
LPC 10%	28.6 ± 0.0 ab	−1.1 ± 0.3 ab	31.5 ± 1.4 abc	9.5	65.8 ± 0.6 bc	10.6 ± 0.2 ab	13.0 ± 0.1 bc	4.7
LPC 15%	29.9 ± 0.0 a	−0.1 ± 0.1 a	32.8 ± 0.9 ab	12.1	65.4 ± 0.8 bc	10.9 ± 0.0 ab	13.0 ± 0.4 bc	7.3

Different letters in columns indicate significant difference (*p* ≤ 0.001; ≤0.01 for a* of crust; ≤0.05 for brown index of crumb).

## Data Availability

The original contributions presented in the study are included in the article/supplementary material, further inquiries can be directed to the corresponding authors.

## Supplementary Materials

The following supporting information can be downloaded at: https://www.mdpi.com/article/10.3390/foods13050807/s1, Table S1: Cluster membership; Table S2: Final cluster centres.
