# Supplementary material for: Lupin as Ingredient in Durum Wheat Breadmaking: Physicochemical Properties of Flour Blends and Bread Quality
Source: Foods. 2024 Mar 6;13(5):807. doi: 10.3390/foods13050807 (PMC10931101; doi:10.3390/foods13050807)
Supplement: Supplementary file 1 [file foods-13-00807-s001.zip › foods-2876077-supplementary.pdf]

Table S1. Cluster membership.

Cluster Membership

Case Number	SAMPLE	Cluster	Distance
1	SC	1	.
2	LPC 3%	2	0,000
3	LPC 7%	3	3,209
4	LPC 10%	3	,791
5	LPC 15%	3	3,273
6	LF 3%	4	2,685
7	LF 7%	4	1,975
8	LF 10%	4	1,135
9	LF 15%	4	3,327

Table **S2.** Final cluster centres.

Final Cluster Centres

	Cluster			
	1	2	3	4
Zscore: Flour_Polyphenols (mg GAE/g d.m.)	-1,11685	-1,09166	-,60671	1,00716
Zscore: Flour_Antioxidant activity (µmol/g d.m.)	-1,34407	-,82868	-,45981	,88805
Zscore: Flour_WBC	-2,02182	-1,35283	,29733	,62067
Zscore: Flour_OBC	2,38273	,86645	-,37907	-,52799
Zscore: Flour_Dough development time (min)	-,82712	-,44397	-,38923	,60970
Zscore: Flour_Stability (min)	,86927	1,16424	,25698	-,70112
Zscore: Flour_Softening degree (B.U.)	-1,39713	-,90968	-,25339	,76675
Zscore: Flour_Water absorption at 500 B.U. (%)	-1,30327	-,52131	-,49959	,83083
Zscore: Flour_W	2,10711	1,13494	-,15241	-,69621
Zscore: Flour_P/L	-,71791	-,56346	-,13443	,42116
Zscore: Flour_Falling number	-1,89917	-1,14950	,51763	,37394
Zscore: Bread_Moisture (g/100g)	1,37251	,94597	,42844	-,90095
Zscore: Bread_Specific volume (cm3/g)	2,39209	,71157	-,60559	-,32172
Zscore: Bread_Density (g/cm3)	-2,36235	-,68384	,62167	,29529
Zscore: Bread_Height (mm)	2,30782	,72986	-,67685	-,25178
Zscore: Bread_Hardness (N)	-,44544	-,59464	-,91067	,94302
Zscore: Bread_Porosity (1-8)*	-1,71660	-1,01436	-,31211	,91682
Zscore: Bread_Polyphenols (mg GAE/g d.m.)	-1,56628	-1,07519	-,32516	,90424
Zscore: Bread_Antioxidant activity (µmol/g d.m.)	-1,95907	-1,05634	,00869	,74734
Zscore: Bread crumb_Brown index (100-L)	-,92934	-1,64583	-,19129	,78726
Zscore: Bread crumb_a*	-1,49561	-1,20854	-,22682	,84615

Zscore: Bread crumb_b*	-1,33437	-1,38766	-,17408	,81107
Zscore: Bread crust_Brown index (100-L)	-,73218	-,99356	-,24151	,61257
Zscore: Bread crust_a*	1,54532	,99264	-,87440	,02131
Zscore: Bread crust_b*	1,64514	1,25655	-,24633	-,54068
